# Supplementary material for: Patterns of Intron Gain and Loss in Fungi
Source: PLoS Biol. 2004 Nov 30;2(12):e422. doi: 10.1371/journal.pbio.0020422 (PMC532390; doi:10.1371/journal.pbio.0020422)
Supplement: Table S1 — Also available at http://genes.mit.edu/NielsenEtAl/. (4.3 MB ZIP). [file pbio.0020422.st001.zip › NielsenEtAl/html/1215.html]

AN0433.1.NCU08389.1.MG03653.1.FG01516.1


```
 CLUSTAL W (1.82) Multiple Sequence Alignments - Introns Inserted


Sequence 1: NCU08389.1	210 aa
Sequence 2: MG03653.1	174 aa
Sequence 3: FG01516.1	161 aa
Sequence 4: AN0433.1	174 aa
Alignment Length: 210 aa
Number Identitical Residues: 107 aa
Alignment Score (without introns) 5109


MG03653.1 	------------------------------------MG1RLQEYQVIGRHLPTESNPTPS
NCU08389.1	MSPNKHTRIFFLACPLIRDILQLNRTKFSRYLATNNHS1RLQEYQVIGRHLPTESNPTPA
FG01516.1 	--------------------------------------~-----------MPTEADPTPA
AN0433.1  	------------------------------------MG1RLTEYQVIGRHLPTEANPTPK
          	                                     .    . .  .  :***::*** 

MG03653.1 	LYRMRIFAPNEVVAKSRYWYFLRGLKKVKKATGEIVAVNK0IHEKHPLKVKNFGIWIRYN
NCU08389.1	LYRMRIFAPNAVVAKSRFWYFLRGLRKVKKATGEIVTCNV0ITEKHPLKVKNFGVWIRYD
FG01516.1 	LYRMTIFAPNETVAKSRYWYFLRGLKKVKKATGEIVSVKV0LHEKHPLKVKNFGVWIRYD
AN0433.1  	LYRMRIFAPNAVVAKSRFWYFLTQLRKVKKANGEIVSLNV0IHEKRPLKVKNFGIWLRYD
          	**** ***** .*****:****  *:*****.****: :  : **:********:*:**:

MG03653.1 	SRSGTHNMYKEYRELSRTAAVEAMYQDMAARHRARFASIH0ILRVVELEKTDDIKRPYIK
NCU08389.1	SRSGTHNMYKEYRELSRTAAVEALYSDMAARHRARFRSIH0ILKVVELEKTEDIKRPYLK
FG01516.1 	SRSGTHNMYKEYRELSRTDAVESLYSDMAARHRARFRSIH0VLRVVEIEKTEDIKRPYIR
AN0433.1  	SRSGTHNMYKEFREMSRTEAVEALYQDMAARHRARFGSIH0ILKVVEIEKADSIRRPYIK
          	***********:**:*** ***::*.********** *** :*:***:**::.*:***::

MG03653.1 	QLTTKGLTFPLPHRVPKINNKKIFSARRPSTFA
NCU08389.1	QLVTKKLSFPLPHRVPKISNKKIFSAKRPATFA
FG01516.1 	QLTQKNLSFPLPHRITKENTQKLFSAKRPSTFA
AN0433.1  	QLLQKNLKFPLPHRSATGNKKKVFAYSRPSTFA
          	**  * *.****** .. ..:*:*:  **:***
```
